# Plagiocephaly and brachycephaly treatment with cranial orthosis: a case report

**DOI:** 10.1590/S1679-45082013000100021

**Published:** 2013

**Authors:** Gerd Schreen, Carolina Gomes Matarazzo

**Affiliations:** 1 Clínica Cranial Care, São Paulo, SP, Brazil

**Keywords:** Plagiocephaly, Plagiocephaly, nonsynostotic, Skull/abnormalities, Sudden infant death, Case reports

## Abstract

The number of cranial deformities has increased considerably since international efforts of pediatricians to recommend parents putting their babies to sleep in the supine position as a strategy to reduce sudden death syndrome of the newborn. On the one hand, this program has demonstrated very efficient results at reducing deaths and, on the other hand, such recommendation has increased the incidence of cranial asymmetries. In addition, infants are kept too long in one position, much of this due to abusive use of strollers, baby carriers, car seats, swings and other devices. Among resulting asymmetries, the most frequently found are plagiocephaly (parallelogram shaped skull, with posterior unilateral flattening with the opposite frontal area also flattened) and brachycephaly (occipital bilateral flattening). The present study is a case report of a patient with brachycephaly associated with deformational plagiocephaly treated with cranial orthosis. The same physician clinically evaluated the patient before and after treatment using photographic recording and a laser scanning device, which allows the accurate measurement of variables determining asymmetries. It became clear during treatment that there was significant improvement in cranial symmetry documented by decrease in the cephalic index, diagonal difference and volume gain in the quadrant that was flattened. The authors conclude that orthotic therapy is a safe and effective therapeutic modality for position cranial asymmetries.

## RESUMO

O número de deformidades cranianas tem aumentado desde que tiveram início os esforços internacionais dos pediatras, com a recomendação de se colocar os filhos para dormirem na posição supina, como estratégia para reduzir a morte súbita do recém-nascido. Se, por um lado, esse programa conseguiu demonstrar resultados muito eficientes nessa redução, por outro, tal recomendação fez com que os casos de assimetrias cranianas aumentassem em incidência. Isso porque os lactentes são mantidos por muito tempo em um só posicionamento, pois há também o uso abusivo de dispositivos como carrinho, bebê-conforto, cadeirinha de carro, balancinho, entre outros. Entre as assimetrias resultantes, as mais encontradas são a plagiocefalia (o crânio em forma de um paralelograma com achatamento occipital e anterior contralateral) e a braquicefalia (o achatamento occipital bilateral). Esse estudo relatou o caso de paciente com uma braquicefalia associada à plagiocefalia deformacionais tratado com órtese craniana. O paciente foi avaliado antes e após o tratamento clinicamente pelo mesmo médico, por meio de registro fotográfico e de um escaneamento a laser, que permite aferir variáveis determinantes das assimetrias. Foi possível, durante o período de tratamento, observar que houve importante melhora na simetria craniana documentada pela diminuição do índice cefálico, diminuição da diferença diagonal e ganho de volume no quadrante que se encontrava mais achatado. Conclui-se que a terapia ortótica constitui modalidade terapêutica segura e eficaz disponível para o tratamento das assimetrias cranianas posicionais.

## INTRODUCTION

The number of cranial deformities has grown since international efforts of pediatricians recommending parents to put their babies to sleep in the supine position as a strategy to reduce sudden death syndrome of the newborn. The program has been able to achieve very efficient results and it is known to have reduced cases by 40% in the United States. However, the association between the sleeping position adopted and the several baby accessories increasingly used by parents, such as strollers, swings, car seats, baby carriers and other devices, has helped decrease the time children remain in the prone position, which may contribute to the development of cranial asymmetry, given the extensive use of these accessories can potentially deform the skull^(1–3)^.

Deformational plagiocephaly is a skull asymmetry resulting from external forces applied to an infant´s malleable skull, and its most common presentation is the parallelogram, with occipital flattening, an anterior ipsilateral caput succedaneum, and contralateral occipital bulging. Brachycephaly, in turn, is known to have the same etiology and refers to bilateral occipital flattening^(4–8)^.

The extrinsical factors responsible for infants´ skull deformities, have already been well documented and may begin *intra uterus*, involving several aspects: a very large fetus, multiple gestations, very small maternal pelvis, a small or malformed uterus, excess or scarcity of amniotic fluid, and even increased abdominal muscle tonus may be determining restrictive factors^(3,4)^. Most skull deformities present at birth resolve approximately six weeks after delivery, once the deforming force is removed, although it is important to understand that if these forces persist, deformities may not regress, sustaining asymmetries, such as plagiocephaly and a brachycephaly^(5,6)^. Most cases, however, develop throughout the initial months of life, from a normal skull at birth.

The diagnosis of cranial deformities is clinical, and it is important that the pediatrician include, during the examination of the baby´s head, visualization from the incidence in which the parallelogram is more easily seen. Imaging is kept to the investigation of other presumptive diagnoses, such as craniostenosis, in case of questions as to the etiology of the deformity^(9)^.

The most advanced centers for treatment of cranial asymmetries currently have noninvasive scanner technology that allows attaining a 3-D skull image, without using ionizing radiation or anesthesia. Class I laser (therefore, safe for eyes), from four sources distributed around the skull circumference, uses eight cameras and the image is rebuilt by specific software, resulting in very accurate measurement and indexes. The same measurements can be repeated throughout follow-up, enabling their comparison throughout time. Additionally, the virtual template obtained by scanning can be used to make the customized cranial orthesis, that avoids submitting the child to the uncomfortable cast template molding process^(9,10)^.

In most cases, therefore, the cause of deformities is the fact that infants remain in a single resting position.

If the deformity is detected early (before 3 months of age), repositioning may reach an effective result and be tried up to the 5^th^ or 6^th^ month of life. The choice of treatment modality is an active area of research^(5)^.

Treatment with skull orthesis has been described as safe, without interfering in the growth of the head circumference, and has been increasingly used, since it was first documented in 1979, by Claren^(11)^. There is already evidence, that this orthotic treatment yields statistically superior results in improving asymmetry in comparison to active repositioning^(12–14)^.

The objective of the present study was to demonstrate the result attained during treatment with a cranial orthesis (helmet) for a patient diagnosed as brachycephaly associated with deformational plagiocephaly.

## CASE REPORT

Patient MMB, 5 month-old, male full-term baby was brought by parents for assessment of cranial asymmetry, given they had noticed occipital flattening at the age of 3 months. Since then, the parents had made efforts to reposition the baby to improve the shape of the skull, albeit unsuccessfully. The patient was assessed on April 8, 2011 by physical examination, photo registration using a Nikon™ camera and 3-D laser scanning with Star Scanner Acquisition System™ equipment (Orthomerica Inc., Orlando, FL)^(10)^.

Upon physical examination, there was an important flattening of the child´s entire occipital region, more prominent on the left, and the left ear anteriorization in relation to the right one. No limitation of the child´s cervical range of movement was detected while following visual and auditory stimuli which could suggest congenital torticollis. Likewise, there was no clinical evidence to suspect of craniostenosis.

The baby was then positioned on an appropriate chair, and right side and vertex incidences pictures were taken. On the same occasion a scan was performed.

The scanning process uses anatomical reference points to determine a reference plane from which anthropometric measurements are obtained, the skull is divided into four quadrants, and volume indexes are calculated. The main anatomical landmarks are the selion and the right and left tragions (upper margin of ear tragus). Interference from hair is eliminated using a stockinet made of white malleable fabric.

Data obtained was analyzed using STARscanner Comparison Utility software and sent electronically to Orthomerica Products Inc. (Orlando, FL, USA), which, in turn, manufactured an accurate template with the same shape of the child's head. Such template allowed a customized orthesis to be made, which began to be used on May 2, 2011, when initial adjustments were made and orientation was provided on how to use the device. The main orientation given referred to the recommended period of utilization (23 hours a day), adaptation period and how to clean the orthesis (only with 70% alcohol, daily). In addition, information on warning signs of exaggerated pressure points was given.

From beginning to end of treatment, there were 6 follow-up visits with two-week intervals approximately, on which adjustments were made on the orthesis, to adapt to skull growth, and direct growth toward desired sites.

After one month of treatment, photo registration and scan were repeated to monitor development, and also upon discharge on July 29, 2011, in order to document results. These periods were named T1 (initial assessment and scan), T2 (reassessment for follow-up, one month after initiating use of the orthesis) and T3 (last assessment made, totaling 3 months and 3 weeks from initial assessment).

**Figure 1:**
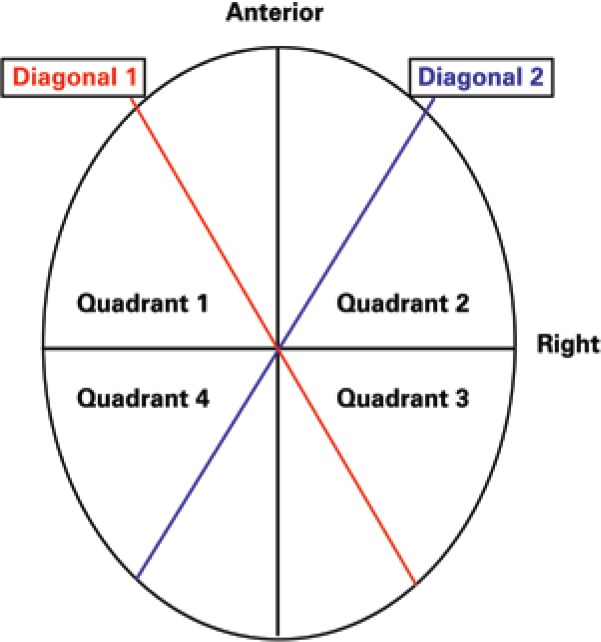
Representation of cross-section of quadrants

The values obtained before and after treatment were compared and the following variables were used:

– cephalic index: percentage resulting from dividing skull width by skull length at level 3 (plane 3cm above the reference plane);
– quadrant volume: measurement of the volume (in cm^3^) of each quadrant, from level 2 to level 8 of the cross section of the child´s head, as shown in figure 1;
– diagonal difference: difference, measured in mm, among 30° oblique diagonals;
– cranial asymmetry index: difference between diagonals 1 and 2, at 30°, divided by the largest diagonal, expressed in percentage;
– anterior symmetry ratio: index obtained from the division of the smallest anterior quadrant by the largest anterior quadrant, resulting in a comparative percentage between both;
– posterior symmetry ratio: posterior percentage index, analogous to the anterior symmetry ratio^(10)^.

**Figure 2:**
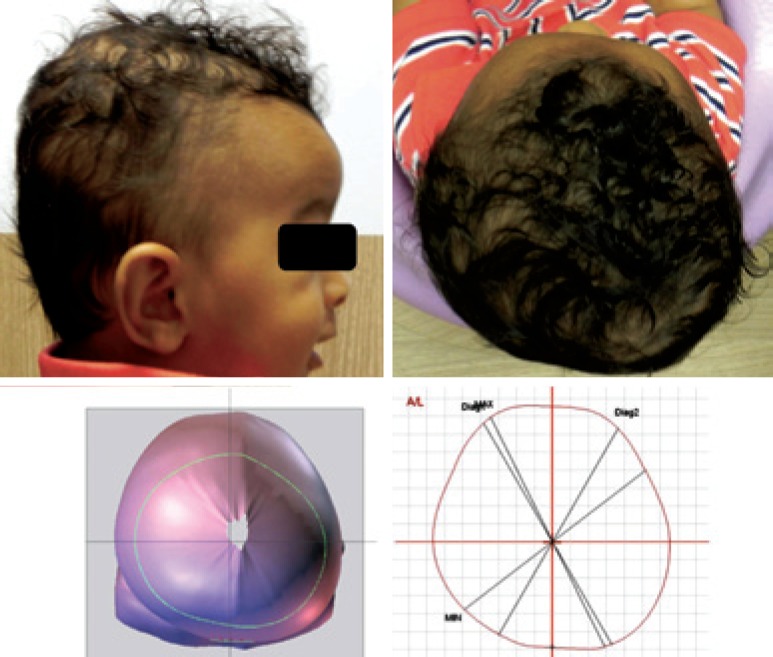
Right side image and vertex of the infant and result of the initial scan, showing the asymmetrical flattening of the occipital region

Figure 2 shows the initial photograph and scan. Based on the image, the flattening of the entire occipital region is evident, larger on the left side, and with slight right frontal flattening.

**Figure 3:**
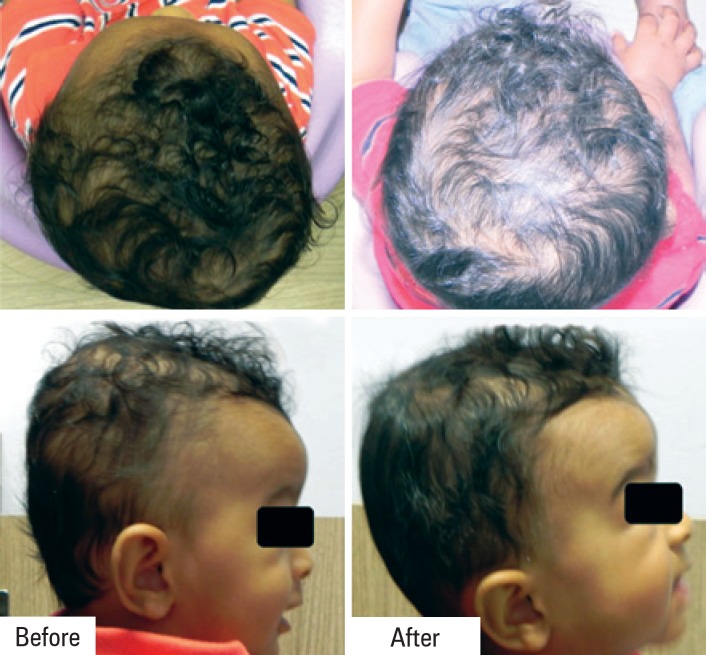
Comparison before and after 3 months of orthesis

Eight and sixteen weeks after the initial scan and totaling, respectively, one and three months of treatment, the clinical exam did not show any asymmetry. Figure 3 shows the images obtained on the last assessment, after three months of treatment.

Table 1 shows data obtained before beginning treatment and after 3 months and 3 weeks of assessment, totaling a 3-month management period. The high value of the cephalic index on the initial evaluation and of the diagonal difference should be pointed out, and anterior and posterior symmetry ratio significantly lower than 1 (1 or 100% would represent a perfect symmetry between sides). Likewise, improvement of all indexes on the second scan stand out in the analysis.

**Table 1:** Data from the initial scan (T1), and 3 months and 3 weeks after (T3)

Measurement	T1	T3
Cephalic index (LL/AP)	0.97	0.89
Diagonal difference (mm)	10.0	1.2
Cranial asymmetry index	7.0	0.8
Anterior symmetry ratio	0.924	0.938
Posterior symmetry ratio	0.902	0.938

Table 2 shows the more significant skull growth on the region in which the orthesis allowed growth, presenting the increase in volumes, which was more pronounced on the posterior region, mainly to the left.

**Table 2:** Data from the initial assessment scan (T1) and 3 months after (T3)

Volumes	T1	T3
Volume quadrant 1	233.3	238.0
Volume quadrant 2	215.6	219.1
Volume quadrant 3	196.1	228.7
Volume quadrant 4	176.9	243.8

**Figure 4:**
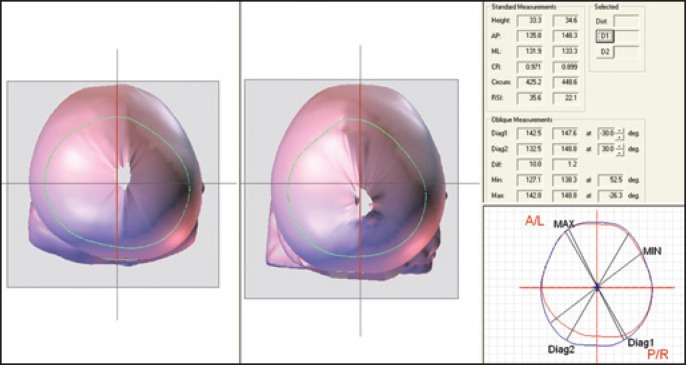
Comparison of image of initial and final scan with 3D view of vertex and of cranial circumference before treatment (in red) and after 3 months using orthesis (in blue), showing the growth in the desired region, quantified by distance D1 and by increased volume in quadrant 4

Comparative analysis of scans on T1 and T3 is shown in figure 4.

## DISCUSSION

As shown by the results presented, there was a major improvement in cranial symmetry, documented by decrease in the cephalic index, translating a better ratio between infant's skull length and width. This parameter, as demonstrated by Plank et al.^(10)^, is one of the main variables to asses skull disproportions such as brachycephaly.

Ratios or indexes are essential to assess plagiocephaly, as they determine reproducible comparisons from one subject to another, regardless of patient's age or head circumference.

The increase in quadrant 4 is easily observed in figure 4, area in which the growth was desired most, to reach better posterior symmetry of the head. The consequent increase in volume of this quadrant, in relation to the remaining ones, can be checked on table 2, and also the largest posterior symmetry index.

These results occur due to the constant contraposition that the orthesis offers to protuberant anterior and posterior regions, in which growth is not desired, while simultaneously allowing free space for the flattened regions to reach the desired growth. In this way, the orthesis conducts and shapes the natural growth of the infant's skull. Frequent adjustments to the orthesis are necessary, typically every 15 days, during the entire treatment, occasions on which the result of correction is also supervised.

Other studies have already showed major decreases in the value of the cranial asymmetry index with orthesis treatment. Mulliken et al.^(14)^ concluded that the treatment with a helmet has determined better statistically significant reduction in this index in comparison to active repositioning. Graham et al.^(13)^ and Rogers et al.^(15)^ have also documented the same finding in their studies.

Graham et al.^(13)^ also considered that the diagonal difference should be less than 3 mm, for the symmetry to considered “normal”. In the treatment described, the diagonal difference went from 10 to 1.2 mm, that is, with the increase in the area of the fourth quadrant, the diagonal difference at 30° was brought to normal indexes.

It is important to present the results that have been achieved in clinical practice, since there are no Brazilian data regarding treatment with skull ortheses. Such technique has been investigated and used in the United States for over two decades, and the scanning technology has only been available in our country recently. This scenario makes the case report important, and also stimulates domestic scientific production, very scarce in this area^(12,13)^.

Deformational plagiocephaly is not a progressive condition like craniostenosis. However, severe cases may cause serious emotional and psychological problems, and concerns about the patients' perception of self-image. To avoid this, early and appropriate instructions to keep the baby in the supine position to reduce risk of sudden death of the newborn, along with orientation to change the baby's position when awake and that it play in the prone position under supervision, are important, avoiding therefore, more pronounced cases of positional plagiocephaly. Attention should be equally paid to early diagnosis of congenital torticollis and other conditions that favor the vicious support on one of the sides of the head.

The treatment of this condition requires joint effort of parents and pediatricians, who should assess the shape of the infant's skull early, ideally before 2 months of age, and, if there is an association with congenital torticollis, physical therapy should be immediate. When parents find information on deformational plagiocephaly and act immediately, stimulating repositioning, it is possible to correct deformity with conservative and low cost treatment, in most cases. Several studies, however, have already clearly established guidelines for the treatment approach to be changed at the right moment, without unjustified delay in using skull orthesis, whenever indicated. Such studies recommend that babies with moderate to severe plagiocephaly be treated with customized ortheses^(11,14,16,17)^, and the present case report underscores the results obtained with orthotic treatment.

## CONCLUSION

Orthotic treatment is a safe and effective treatment modality, and when appropriately indicated, shapes the skull to the desired symmetry, shown by the undeniable improvement in all measurements, and symmetry indexes and variables. The authors emphasized the need for domestic studies that may show the statistically supported results observed in the treatment, comparing it to other therapeutical modalities and to the international literature.
